# Association between Dietary Patterns during Pregnancy and Children’s Neurodevelopment: A Birth Cohort Study

**DOI:** 10.3390/nu16101530

**Published:** 2024-05-19

**Authors:** Jiajun Ouyang, Wenjin Cai, Penggui Wu, Juan Tong, Guopeng Gao, Shuangqin Yan, Fangbiao Tao, Kun Huang

**Affiliations:** 1 School of Public Health, Anhui Medical University, No 81 Meishan Road, Hefei 230032, China; oyjj1925@163.com (J.O.); sdcaiwenjin@163.com (W.C.); wupenggui@hotmail.com (P.W.); m15755162284@163.com (J.T.); ggpahmu@126.com (G.G.); shuangqinyan@126.com (S.Y.); fangbiaotao@163.com (F.T.); 2 Key Laboratory of Population Health Across Life Cycle, Ministry of Education of the People’s Republic of China, Anhui Medical University, No 81 Meishan Road, Hefei 230032, China; 3 NHC Key Laboratory of Study on Abnormal Gametes and Reproductive Tract, No 81 Meishan Road, Hefei 230032, China; 4 Anhui Provincial Key Laboratory of Environment and Population Health across the Life Course, No 81 Meishan Road, Hefei 230032, China; 5 Maternal and Child Health Care Center of Ma’anshan, No 24 Jiashan Road, Ma’anshan 243011, China

**Keywords:** pregnancy, dietary pattern, children, neurodevelopment

## Abstract

Background: Research studies have showed that maternal diet may influence fetal neurodevelopment, but most studies have only assessed single nutrients or food groups. Objective: To investigate the impact of maternal prenatal dietary patterns during pregnancy on child neurodevelopment. Methods: Study participants were obtained from the China National Birth Cohort. The Ages and Stages Questionnaire, Third Edition, was used to assess children’s neurodevelopment at 36 months old. Maternal antenatal dietary data were collected over three trimesters using food frequency questionnaires. Five distinct maternal dietary patterns throughout pregnancy were identified by principal component analysis, namely protein- and micronutrient-rich dietary patterns, low-iron dietary patterns, pasta as the staple food dietary patterns, iron-rich dietary patterns, tubers, fruits, and baked food dietary patterns. Group-based trajectory modeling was performed for dietary patterns present in all three periods. Multiple linear regression models were used for statistical analysis. Results: Children of mothers who followed a high protein- and micronutrient-rich dietary pattern trajectory during pregnancy presented better neurodevelopment, including higher gross motor and problem-solving scores. Furthermore, it was observed that children born of women with low-iron dietary patterns had poorer neurodevelopment. In detail, children born to mothers with a low-iron dietary pattern during the first trimester had lower problem-solving scores, while to those who were exposed to a low-iron dietary pattern in the second and third trimesters had lower gross motor scores. Additionally, children with mothers who had a low-iron dietary pattern in the third trimester had lower communication scores. Conclusions: A nutrition-balanced protein- and micronutrient-rich dietary pattern and adequate iron dietary pattern for mothers throughout pregnancy may be beneficial to children’s neurodevelopment.

## 1. Introduction

The Developmental Origins of Health and Disease (DOHaD) theory states that an individual’s nutritional status during critical periods of life (in utero and infancy) may impact their health status later in life [1]. Environmental factors, particularly nutritional factors, are recognized as essential influences on neurodevelopment in early life [2,3,4]. A growing body of evidence suggests that maternal nutrition during pregnancy may affect offspring’s cognitive development and behavior [5,6,7].

Most previous researchers focusing on maternal nutrition and children’s neurodevelopment have focused on the effects of a single nutrient or food group in pregnant women. For instance, maternal deficiencies in nutrients such as iron or iodine not only affect fetal development but also cause damage to children’s intelligence and cognition [8]. Folic acid supplementation during pregnancy helps prevent neural tube defects and has been shown to improve cognitive development [9]. However, the daily diet of individuals in the modern world often consists of various foods or nutrients, and analytical methods based on a single food or nutrient need to consider the interactions between different types of foods or nutrients. Dietary patterns provide a more comprehensive picture of the nutritional status of women during pregnancy.

The potential influence of maternal dietary patterns during pregnancy on the child’s neurodevelopment has been studied less, and the results vary depending on the difference in how the dietary pattern is defined. In the ALSPAC cohort study by Freitas-Vilela et al., children born of pregnant women in the “vegetables and fruits” group had higher intelligence quotient (IQ) scores compared to the “meat and potatoes” and “white bread and coffee” groups [10]. This is the first study to describe the association between food clusters during pregnancy and childhood IQ. A study of a birth cohort in Jiangsu province, China, showed that the “fish, shrimp, vegetables and algae” dietary pattern of pregnant women during pregnancy may influence the level of neurodevelopment of the offspring through the modulation of downstream metabolites. Concerning women with high scores on this dietary pattern, their infants showed a reduced risk of cognitive and gross motor developmental delays [11]. Maternal adherence to the Mediterranean dietary pattern (MD) during pregnancy was reported to be associated with a decreased risk of neurodevelopmental deficits in their children [12]. Conversely, the higher a woman’s score on unhealthy dietary patterns during pregnancy, the higher the risk of adverse neurodevelopmental outcomes in her offspring. The Generation R study showed that high adherence to the traditional Dutch diet during early pregnancy was associated with an increased risk of externalizing problems in children [13]. In addition, the offspring of mothers following ‘low health’ or ‘high Western’ dietary patterns were predisposed to symptoms of hyperactivity–inattention [14].

Several studies have modeled longitudinal dietary trajectories across the antenatal and postnatal periods [15,16]. These investigations have assessed the stability of diet over time by converting the continuous dietary indicator into an ordered categorical variable at each assessment point [16] or applied latent class transition analysis [15]. However, even less is known about the association between dietary trajectories during pregnancy and child development.

So far, reports on the association between maternal diet quality during pregnancy and childhood development in low- and middle-income countries are lacking. In this study, based on a China birth cohort, we prospectively collected food frequency data using the Food Frequency Questionnaire (FFQ) to investigate the association between dietary patterns in the three trimesters of pregnancy and dietary trajectories during pregnancy and children’s neurodevelopment.

## 2. Methods and Materials

### 2.1. Study Population

**Figure 1:**
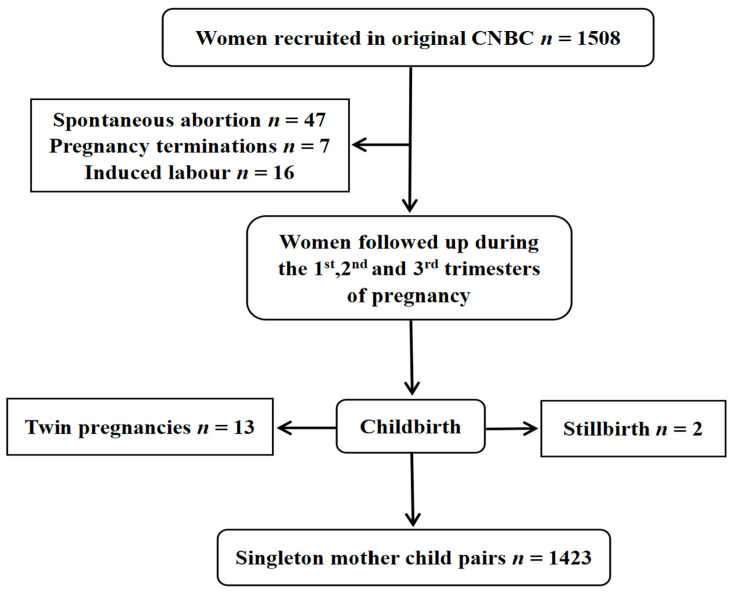
Flowchart of participant recruitment.

This study was based on the China National Birth Cohort (CNBC). Couples in early pregnancy who came for their first antenatal checkup at Ma’anshan Maternal and Child Health Center in Anhui Province, China, from May 2017 to September 2018 were recruited. The inclusion criteria were as follows: ① maternal age ≥ 18 years; ② spontaneous pregnancy; ③ planning to have routine obstetric checkups and delivery at the Center; ④ willingness to be followed up. Notably, women were included in the study regardless of their parity. Initially, a total of 1508 families were enrolled in the cohort. After excluding adverse pregnancy outcomes and twin births, 1423 families remained for inclusion in the study. The flowchart of the participants is shown in Figure 1.

### 2.2. Dietary Assessment

The Food Frequency Questionnaire (FFQ) was used to collect data on the frequency of 26 foods in the first, second, and third trimesters, respectively [17]. In the questionnaire, the pregnant woman reported how often she currently consumed a certain type of food: (1) hardly at all, (2) 1–3 times per month, (3) 1–3 times per week, (4) 4–6 times per week (5) every day. A score of 0, 1, 2, 3, and 4 was assigned to each of the five options, respectively.

To fill in the missing data on the FFQ, we utilized the SPSS Version 26.0 to perform multiple interpolations (MIs) on the data in each trimester of pregnancy, which was a flexible method to impute missing values [18,19]. It compensated for the shortcomings of single interpolation by modeling the likelihood distribution of the missing data so as to reduce the impact of the missing data on the modeling results without distorting them. Finally, five interpolated datasets were generated, and we chose the dataset with the largest Cronbach’s alpha coefficient (Alpha) for analysis.

### 2.3. Food Pattern Determination

From the dietary data collected by the FFQ, we excluded three categories of food, namely spicy food, puffed food, and candy sweets, because they are not part of the typical diet in daily life, and the frequency of their consumption is low. The remaining 24 food items were retained for analysis.

We used principal component analysis to extract dietary patterns during three trimesters of pregnancy. The applicability of factor analysis was first determined based on the KMO statistic and the Bardett spherical test. The KMO values of the first, second, and third trimesters of pregnancy were all greater than 0.5 (0.774, 0.717, and 0.706, respectively), and the *p*-values of Bardett’s spherical test were all less than 0.05, which indicated that the data were able to be processed by factor analysis. Three dietary patterns were identified in each period based on the gravel plot characteristic root (λ > 1.4). The greater the absolute value of the factor loading of a single food entry, the greater the contribution of that food to the principal components. Food entries with an absolute value of the factor loading greater than 0.3 were retained in this study (Supplementary Table S1). Individual dietary patterns were named according to the food components with high contributions to the dietary pattern, thus simplifying the factor structure and increasing interpretability. For each food item in a certain dietary pattern, a score was obtained by multiplying the standardized values of an individual food item’s dietary frequency with the weight of the corresponding principal component (i.e., the absolute value of the factor loading). The overall score of each dietary pattern was the sum of all scores of the food items that were involved in this pattern. After completing the extraction of dietary patterns, each woman would receive a principal component score for each pattern in three trimesters of pregnancy. High scores indicated that the woman favored the dietary pattern.

Three dietary patterns extracted in the first trimester were protein- and micronutrient-rich dietary patterns, low-iron dietary patterns, and pasta as the staple food dietary pattern. Three patterns in the second trimester were the protein- and micronutrient-rich dietary pattern, iron-rich dietary pattern, and low-iron dietary pattern, and the three patterns in the third trimester were the protein- and micronutrient-rich dietary pattern, tubers, fruits, baked food dietary pattern, and low-iron dietary pattern. Protein- and micronutrient-rich dietary patterns and low-iron dietary patterns were involved in all three trimesters.

### 2.4. Evaluation of Children’s Neurodevelopment

The Ages and Stages Questionnaire, Third Edition, (ASQ-3) was adopted to evaluate children’s neurodevelopment at the age of 36 months. It is a widely used and validated parent-completed questionnaire for screening children’s communication, gross motor, fine motor, problem-solving, and personal–social delays [20]. For each item, there were three responses to choose from: “yes”, “sometimes”, and “not yet”, which were scored as 10, 5, and 0. An overall domain of score was then obtained from the sum of all items in each domain. The maximum total scores were then obtained from the sum of all items, with a maximum score of 60. The higher the score, the better the skills and abilities in the given domain. According to the ASQ system of Shanghai Zhangyuan Information Technology Company, the criteria for children who are well-developed in different dimensions were scores of “>50” in the communication and gross motor dimension. “>35” in the fine motor dimension, and scores of “>45” in the problem-solving dimension and personal–social dimension. Otherwise, the children were considered to have developmental delays. The scale had high reliability and validity, with test–retest reliability of 0.82–0.94, internal reliability of 0.75–0.92, and calibrated validity of 0.76–0.94 [21].

### 2.5. Covariates

Potential confounders were identified by the relevant literature and directed acyclic graphs (DAG) [22], including maternal age, pre-pregnancy BMI, maternal occupation before pregnancy, family income, education level, parity, maternal smoking, alcohol consumption, anxiety, depression, and pregnancy complications (Supplementary Figure S1).

At the first antenatal visit, information on maternal age, occupation before pregnancy, family income, education level, maternal smoking, and maternal alcohol consumption were collected through a questionnaire. Furthermore, the obstetricians calculated pre-pregnancy BMI by women’s weight and height. Information on maternal pregnancy complications (gestational hypertension/diabetes) and parity were extracted from the medical notes.

Data on the psychosocial status of pregnant women were assessed using the Center for Epidemiologic Studies Depression Scale (CES-D) and Self-Anxiety Rating Scale (SAS). The CES-D consisted of 20 questions to screen for participants with symptoms of depression [23]. It was rated on a scale of 0–3, with a total score ranging from 0 to 60. The higher the score, the higher the level of depression. It has been widely used in pregnant women with a standard cut-off value of ≥16. The SAS consisted of 20 items reflecting subjective anxiety, each scored on a 4-point scale based on the frequency of symptoms [24]. Women with an SAS score of at least 40 were considered to have anxiety symptoms.

Information regarding infants’ gestational week, birth weight, exclusive breastfeeding and complementary food within six months were collected for sensitivity analysis. Information on the gestational week and birth weight were extracted from medical records. Exclusive breastfeeding within six months was collected from questionnaires completed by primary caregivers.

### 2.6. Statistical Analysis

Linear regression models were developed to analyze the associations between the five dietary pattern scores and children’s neurodevelopment during the three trimesters of pregnancy.

**Figure 2:**
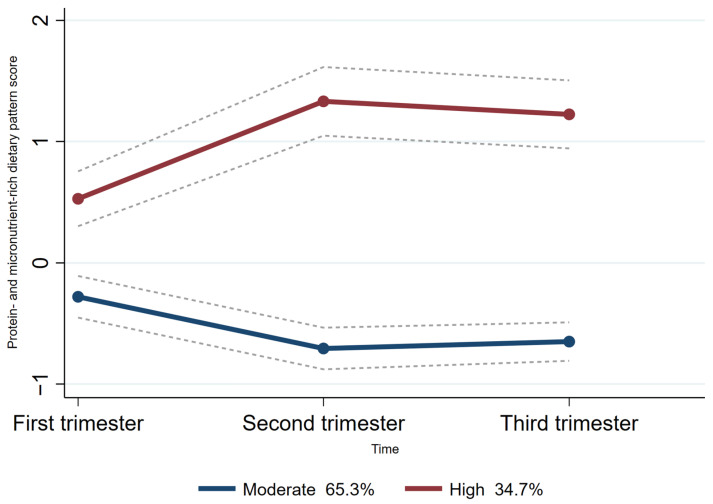
Trajectories of protein and micronutrient-rich dietary pattern score in pregnant women. The dashed lines indicated 95% confidence intervals of trajectories. The circles indicated protein- and micronutrient-rich dietary pattern scores at corresponding observation point in each trajectory group.

**Figure 3:**
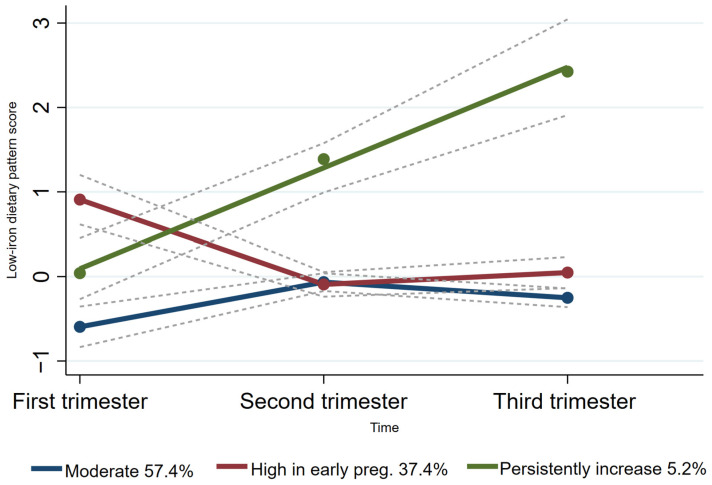
Trajectories of low-iron dietary pattern score in pregnant women. The dashed lines indicated 95% confidence intervals of trajectories. The circles indicated low-iron dietary pattern scores at corresponding observation point in each trajectory group.

Group-based trajectory modeling (GBTM) was used to fit maternal dietary pattern score trajectories according to the individual principal component scores for each dietary pattern across the three trimesters of pregnancy using Stata 15.0 software [25,26]. As the ‘protein- and micronutrient-rich dietary pattern’ and the ‘low-iron dietary pattern’ were the two dietary patterns consistently presented across all trimesters of pregnancy, the trajectories of these two patterns were fitted. The maximum likelihood method was used for parameter estimation and model fitting. Firstly, we explored two to five categories of potential trajectory models, considering existing relevant studies on dietary pattern score trajectories during pregnancy [27,28]. Based on the recommendations [26,29], the detailed selection criteria for the best-fitting model for trajectories were as follows: (1) Bayesian Information Criterion (BIC). The closer the BIC value was to 0, the better the model fit; (2) a higher value of entropy indicated how well the classification distinguishes from one group to another; (3) the average posterior probability was required to be >0.7, and (4) each trajectory contained at least 5% of the participants. Finally, we fitted two categories of protein- and micronutrient-rich dietary pattern scores (moderate trajectories and high trajectories) throughout pregnancy (Figure 2). Additionally, we also fitted three categories of low-iron dietary pattern scores (low trajectories, moderate trajectories, and high trajectories) throughout pregnancy (Figure 3).

Linear regression models analyzed the association between protein- and micronutrient-rich dietary trajectories and low-iron dietary trajectories during pregnancy and children’s neurodevelopment.

Two sensitivity analyses were conducted in this study; Firstly, studies have shown that maternal Mediterranean dietary patterns might have a positive impact on perinatal outcomes, such as being premature and having a low birth weight [30], which in turn were associated with children’s neurodevelopment [31]. Birth weight and gestational age might be the potential mediators in the association between maternal dietary patterns during pregnancy and children’s neurodevelopment. Therefore, we further adjusted for birth weight z-scores in addition to the main analysis.

Second, we recognized that breastfeeding and complementary feeding were not mediating variables but rather were significant postnatal factors influencing children’s neurodevelopment [32,33,34]. As these variables were not regarded as relating to maternal dietary patterns, they served as precision variables and were further adjusted.

The software SPSS Version 26.0 and Stata Version 15.0 were used and a *p*-value of <0.05 was considered statistically significant.

## 3. Results

### 3.1. Baseline Characteristics of Participants

The overall characteristics of the 1423 participants are shown in Table 1. The mean age of the pregnant women included in the study was 28.7 years. The majority of mothers had a body mass index within the normal range. Most of the pregnant women did not smoke or drink alcohol. The median infant’s birth weight was 3353 g, and the average gestational week was 38.9 weeks.

### 3.2. Association between Dietary Pattern Scores during Pregnancy and Children’s Neurodevelopment

**Figure 4:**
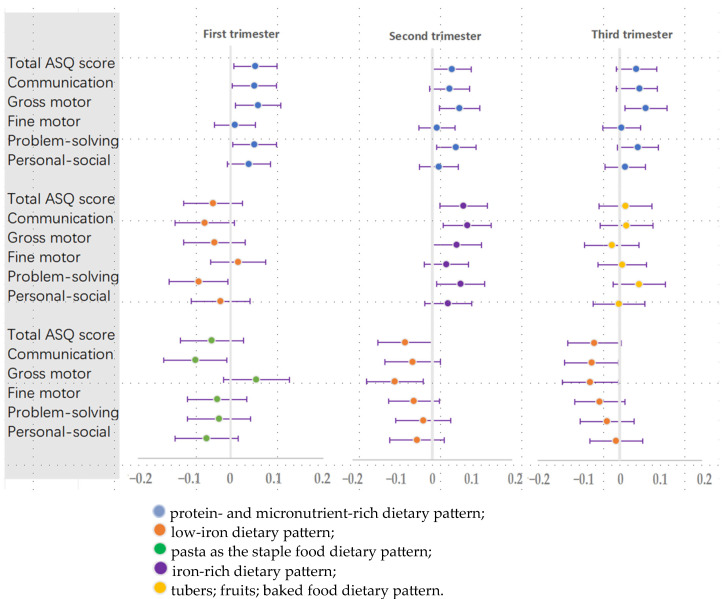
Linear regression between maternal dietary pattern scores during pregnancy and 36-month-old infants’ neurodevelopment [β (95% CI)].

The linear association β (95% CI) between the first, second, and third trimesters’ dietary pattern scores and neurodevelopment scores of 36-month-olds is shown in Table 2 and Figure 4.

In any of the three trimesters of pregnancy, women with high protein- and micronutrient-rich dietary pattern scores had children with better gross motor abilities (β = 0.059, 95% CI: 0.010, 0.108); (β = 0.068, 95% CI: 0.017, 0.119); (β = 0.063, 95% CI: 0.012, 0.114). During the first and second trimesters of pregnancy, women who followed a protein- and micronutrient-rich dietary pattern had children with higher total developmental scores (β = 0.053, 95% CI: 0.006, 0.100); (β = 0.049, 95% CI: 0.001, 0.098), particularly in problem-solving abilities (β = 0.051, 95% CI: 0.004, 0.098); (β = 0.059, 95% CI: 0.010, 0.109). In the first trimester, there was a positive association between the protein- and micronutrient-rich dietary pattern and communication scores (β = 0.051, 95% CI: 0.003, 0.098).

For iron dietary patterns, it was observed that women with low-iron dietary patterns had children with poorer neurodevelopment (first trimester: lower problem-solving scores (β = −0.069, 95% CI: −0.133, −0.006); second and third trimester: lower gross motor scores (β = −0.095, 95% CI: −0.167, −0.023); (β = −0.072, 95% CI: −0.140, −0.004); third trimester: lower communication (β = −0.068, 95% CI: −0.134, −0.003)).

In the second trimester, women with iron-rich dietary pattern scores had children with high total ASQ scores and communication and problem-solving scores (β = 0.078, 95% CI: 0.018, 0.138); (β = 0.088, 95% CI: 0.027, 0.148); and (β = 0.071, 95% CI: 0.011, 0.131).

### 3.3. Characteristics of the Distinct Protein- and Micronutrient-Rich Dietary Pattern Trajectory Groups

For the protein- and micronutrient-rich dietary pattern trajectory, the majority (65.3%) of women followed a “moderate protein- and micronutrient-rich dietary pattern trajectory.” This means this group of women had a moderate protein- and micronutrient-rich dietary pattern score at each time point. Another 34.7% of women followed a “high protein- and micronutrient-rich dietary pattern trajectory”, meaning they had a high protein- and micronutrient-rich dietary pattern score at each period (Figure 2).

### 3.4. Characteristics of the Distinct Low-Iron Dietary Pattern Trajectory Groups

For the low-iron dietary pattern trajectory, the majority (57.4%) of women followed a “moderate” low-iron dietary pattern trajectory and 37.4% of women followed a “high in early pregnancy” low-iron dietary pattern trajectory. Furthermore, only 5.2% of women followed a “persistently increased” low-iron dietary pattern trajectory (Figure 3).

### 3.5. Associations between Trajectories of Women’s Protein- and Micronutrient-Rich Dietary Pattern Scores in the Three Periods of Pregnancy and Children’s Neurodevelopment

As shown in Table 3, after adjusting for potential confounders, children of mothers who had a protein- and micronutrient-rich dietary pattern high-trajectory mothers had a high score of total ASQ (β = 5.107, 95% CI: 0.464, 9.750). Their children had relatively higher gross motor (β = 1.279, 95% CI: 0.289, 2.269) and problem-solving (β = 1.478, 95% CI: 0.221, 2.735) abilities.

### 3.6. Associations between Trajectories of Women’s Low-Iron Dietary Pattern Scores in the Three Periods of Pregnancy and Children’s Neurodevelopment

After adjusting for potential confounders, no statistically significant associations existed between the trajectories of women’s low-iron dietary pattern scores in the three periods of pregnancy and children’s neurodevelopment (Table 3).

### 3.7. Sensitivity Analyses

Sensitivity analyses did not fundamentally change the main results (Supplementary Tables S2 and S3).

## 4. Discussion

Our findings support that mothers’ adherence to a protein- and micronutrient-rich dietary pattern during pregnancy was a protective factor for children’s neurodevelopment, and adherence to a low-iron dietary pattern was a risk factor for children’s neurodevelopment. Further analyses showed that children of mothers who had a high trajectory of protein- and micronutrient-rich dietary patterns had a lower risk of abnormal neurobehavioral development than those with a moderate trajectory.

Gestation is a critical window for development, and mothers should pay more attention to a proper diet during pregnancy to ensure appropriate and adequate nutrient intake. Nutrient deficiencies early in life, despite adequate supplementation later in life, still pose a risk of dysfunction and altered developmental trajectories in adults [35]. Unlike most previous studies that have explored the effects of intake of single or multiple nutrients on neurodevelopment, our results are studies of dietary patterns based on good food sources of a particular nutrient or nutrients. Dietary pattern studies can translate nutrient intake into food services and provide applicable dietary recommendations to the public.

We found that children of mothers who had protein- and micronutrient-rich dietary patterns during pregnancy had a lower risk of abnormal neurodevelopment. Protein is essential for maintaining human growth and development. Maternal protein malnutrition may lead to disrupted brain development in the offspring, with lasting effects on motor and cognitive function [36]. The Japanese Environment and Children Study also found that low protein intake in early pregnancy was associated with a higher risk of developmental delay in 3-year-old children [37]. In rodents, protein malnutrition in early life stages will alter neurogenesis, cell migration, differentiation, and plasticity [38,39,40,41]. The mechanisms involved may be maternal protein restriction, affecting neurotransmitters and hormone release in the hippocampus, cortex, and hypothalamus and neural stem cells’ (NSCs) ability to proliferate and differentiate [41,42,43,44]. In addition to protein, micronutrients such as iodine and iron and deficiencies of vitamin C, folic acid, and other B vitamins at all stages of pregnancy are detrimental to fetal neurodevelopment. Inadequate iodine intake during pregnancy affects thyroid hormone synthesis, which, in turn, affects neuronal proliferation and migration in the cortex and hippocampus of the offspring and impairs fetal brain development [45]. Severe iodine deficiency may lead to intellectual disability in children and is the most common cause of preventable brain damage worldwide [46]. Additionally, vitamin C is vital in fetal brain development during pregnancy. Prenatal vitamin C deficiency has been reported to severely impair hippocampal development and induce oxidative stress in the developing guinea pig brain [47]. The intake status of B vitamins, folic acid, vitamin B12, vitamin B6, and riboflavin may all affect the overall status of choline, which can have a direct impact on fetal neurodevelopment, both prenatally and postnatally, and may also have an effect on methylmercury toxicity [48]. Studies have shown that intake of folic acid and vitamin B12 during pregnancy can help prevent neural tube defects [49]. Our study highlighted that eating food groups containing both proteins and micronutrients throughout the pregnancy benefits the offspring’s neurodevelopmental behaviors. We acknowledge that while a balanced diet containing protein and micronutrients is beneficial for promoting fetal neurodevelopment, it is important to note that studies have suggested a potential risk associated with maternal high-protein diets during pregnancy, i.e., an increased risk of obesity and metabolic syndrome in offspring [50,51]. Therefore, while we encourage pregnant women to maintain a diverse diet that includes protein- and nutrient-rich foods, we also emphasize the importance of moderating protein intake to prevent overconsumption.

We also found that women’s adherence to a low-iron dietary pattern was negatively associated with children’s neurodevelopment. Iron deficiency and iron deficiency anemia are major nutritional disorders worldwide. Early iron deficiency (ID) was associated with altered brain function that supports active and passive cognitive control in childhood [52]. Good prenatal iron levels are essential for proper neurological development in children [53]. Iron is required for enzymes involved in specific brain functions, including myelin formation and synthesis of the neurotransmitters serotonin (tryptophan hydroxylase) and dopamine (tyrosine hydroxylase) and precursors of epinephrine and noradrenaline [54]. Animal experiments have found that the offspring of rats that followed an iron-deficient dietary pattern during pregnancy had less myelin formation in the subcortical white matter and hippocampal hairs in later life than those of rats who prenatally had an iron-sufficient dietary pattern, even though the pups were consumed postnatally on a diet containing sufficient iron [55]. Recent cohort studies have shown that maternal serum ferritin levels (ranging from 12 mg/L to 60 mg/L) and iron intake (ranging from 14.5 mg/day to 30.0 mg/day) are associated with higher scores regarding working memory and executive functioning in offspring, respectively [56]. Our discovery recommended that dietary iron intake be appropriately enhanced during pregnancy. Further studies are warranted to support the findings.

Our results showed that children of mothers who had a high trajectory of protein- and micronutrient-rich dietary patterns had a lower risk of abnormal neurobehavioral development than those with the moderate trajectory. GBTM has emerged as a focal point in recent research for modeling dietary trajectories during pregnancy. By leveraging longitudinal dietary intake data, researchers can employ GBTM to identify distinct dietary patterns and trends among different groups of pregnant women. The method’s advantage lies in its ability to uncover the diversity of dietary habits during pregnancy and identify potentially health-relevant food patterns. Recent studies have highlighted the significance of GBTM in understanding the impact of maternal dietary intake on the health of both pregnant women and their fetuses. However, studies on the association between diet trajectories during pregnancy and offspring neurodevelopment are scarce. To further examine changes in mothers’ dietary patterns during different periods of pregnancy and their associations with children’s neurobehavioral development, we constructed trajectories of the protein- and micronutrient-rich and the low-iron dietary patterns using repeated measures data. Women with moderate protein- and micronutrient-rich trajectories scored relatively high in the first stage, and women with high protein- and micronutrient-rich trajectories scored relatively low in the first stage. Our identified dietary patterns differed from other studies. However, the stability of the dietary pattern trajectories identified in our study is similar to studies reporting dietary changes over time in Chinese adults [57,58]. This approach deserves further replication to strengthen the evidence of dietary changes during pregnancy and their effects.

Our study has several strengths. One of the strengths of dietary pattern studies is the ability to translate nutrient intake into food supply and thus provide applicable dietary recommendations to the public. The results of this study support many of the previous results of many single foods or nutrients and children’s intellectual development. We used a fully validated FFQ to repeatedly collect data in three trimesters of pregnancy, allowing us to capture changes in dietary intake throughout pregnancy. In addition, many studies have begun to fit trajectories of dietary patterns during pregnancy based on data from repeated measures [27,28]. In contrast, almost no studies explore the effects of the trajectories of mothers’ dietary patterns during pregnancy on offspring neurodevelopment, which is the novelty of this paper. Finally, this study was based on a prospective birth cohort, and the data used in the analyses were obtained through a follow-up questionnaire that included exposure, outcome variables, and confounders, effectively reducing recall bias. Accuracy variables affecting neurodevelopment were also considered in sensitivity analyses to improve the accuracy of the findings.

There were, of course, some limitations in this study. We were unable to collect and control for all potential confounders; furthermore, we also did not collect some of the factors that may affect children’s neurodevelopment such as maternal sleeping status in pregnancy and infants’ feeding patterns [34,59]. Particularly, 36.8% infants had received complementary food before 6 months of age. Although further adjustment of this variable did not change the main findings, we still lacked detailed information on the complementary food that children had. This did not allow us to observe which kind of complementary food would or would not impact children’s neurodevelopment. In addition, the dietary patterns identified in this study were based on nutrient food sources and do not accurately represent the extent to which precise levels of intake of nutrients such as protein are associated with outcomes. Moreover, multiple methods should be used to comprehensively assess neurodevelopmental outcomes, such as the Bayley Scale of Infant and Toddler Development, Third Edition. Finally, this study is based on a cohort study in China, with limitations in extrapolating the results to people other than Chinese populations and slight limitations in inferring causality from observational data.

## 5. Conclusions

The study supports that following a protein- and micronutrient-rich dietary pattern during pregnancy was positively associated with neurodevelopment in offspring. The risk of neurodevelopment abnormalities in the offspring increased when women followed a low-iron dietary pattern during pregnancy. 

## Figures and Tables

**Table 1:** Baseline characteristics of participants.

Characteristics	Distributions
Demographic characteristics	
Maternal educational level [*n* (%)]	
Junior high school or below	8 (0.6)
Senior middle school	507 (35.6)
Junior college or above	908 (63.8)
Annual household income (CNY one million) * [*n* (%)]	
<5	192 (13.5)
5~10	698 (49.1)
10~20	412 (29.0)
20~30	95 (6.7)
>30	24 (1.7)
Maternal characteristics	
Maternal age, years (Mean ± SD)	28.7 ± 4.0
Body mass index before pregnancy * [*n* (%)]	
Underweight (BMI < 18.5)	227 (16.0)
Normal (18.5 ≤ BMI < 24.9)	1028 (72.2)
Overweight obesity (BMI ≥ 24.9)	164 (11.5)
Maternal occupation [*n* (%)]	
Brain work	637 (44.8)
Manual work	509 (35.8)
Else	88 (6.2)
No work	189 (13.3)
Smoking during pregnancy * [*n* (%)]	
Yes	30 (2.1)
No	1389 (97.6)
Alcohol during pregnancy * [*n* (%)]	
Yes	352 (24.7)
No	1067 (75.0)
Hypertension during pregnancy * [*n* (%)]	
Yes	13 (0.9)
No	1302 (91.5)
Diabetes mellitus during pregnancy * [*n* (%)]	
Yes	317 (22.3)
No	998 (70.1)
Pregnancy anxiety * (Mean ± SD)	39.5 ± 8.0
Pregnancy depression * (Mean ± SD)	13.7 ± 7.9
Parity * (Mean ± SD)	
0	11 (0.8)
1	574 (40.3)
2	306 (21.5)
3	2 (0.1)
Children’s characteristics	
Gestational weeks * (Mean ± SD)	38.9 ± 1.4
Neonatal weight (g) * (Mean ± SD)	3353 ± 463
Exclusive breastfeeding for 6 months * [*n* (%)]	
Yes	633 (44.5)
No	603 (49.4)
Complementary food within 6 months * [*n* (%)]	
Yes	524 (36.8)
No	845 (59.4)

* Missing data: 2 in annual household income; 4 in body mass index before pregnancy, smoking, alcohol during pregnancy; 108 in hypertension, diabetes mellitus during pregnancy; 187 in exclusive breastfeeding for 6 months; 54 in complementary food within 6 months; 5 in pregnancy anxiety, depression; 111 in gestational weeks and neonatal weight; 530 in parity. Abbreviations: BMI, body mass index; SD, standard deviation.

**Table 2:** Linear regression analysis between maternal dietary pattern scores during pregnancy and 36-month-old infants’ neurodevelopment [β (95% CI)].

Maternal Dietary Pattern	Model	Total ASQ Score	Communication	Gross Motor	Fine Motor	Problem-Solving	Personal-Social
**First trimester**							
Protein- and micronutrient-rich	**1**	0.035 (0.005, 0.065)	0.041 (0.011, 0.072)	0.021 (−0.010, 0.051)	0.015 (−0.015, 0.045)	0.033 (0.002, 0.063)	0.021 (−0.009, 0.051)
	**2**	**0.053** **(0.006, 0.100)**	**0.051** **(0.003, 0.098)**	**0.059** **(0.010, 0.108)**	0.009 (−0.035, 0.053)	**0.051** **(0.004, 0.098)**	0.039 (−0.007, 0.086)
Low-iron	**1**	−0.042 (−0.083, 0.001)	−0.056 (−0.097, −0.014)	−0.033 (−0.074, 0.009)	−0.011 (−0.053, 0.030)	−0.062 (−0.104, 0.021)	−0.003 (−0.045, 0.039)
	**2**	−0.038 (−0.102, 0.025)	−0.056 (−0.120, 0.008)	−0.035 (−0.102, 0.031)	0.016 (−0.043, 0.075)	**−0.069** **(−0.133, −0.006)**	−0.022 (−0.085, 0.041)
Pasta as the staple food	**1**	−0.028 (−0.074, 0.017)	−0.033 (−0.079, 0.012)	0.006 (−0.040, 0.052)	−0.021 (−0.067, 0.025)	−0.009 (−0.055, 0.036)	−0.035 (−0.081, 0.011)
	**2**	−0.041 (−0.109, 0.027)	**−0.076** **(−0.145, −0.008)**	0.055 (−0.016, 0.126)	−0.029 (−0.093, 0.035)	−0.025 (−0.094, 0.043)	−0.052 (−0.120, 0.016)
**Second trimester**							
Protein- and micronutrient-rich	**1**	0.021 (−0.012, 0.054)	0.017 (−0.016, 0.050)	0.025 (−0.008, 0.059)	0.006 (−0.027, 0.040)	0.034 (0.001, 0.067)	0.001 (−0.033, 0.033)
	**2**	**0.049** **(0.001, 0.098)**	0.043 (−0.007, 0.093)	**0.068** **(0.017, 0.119)**	0.011 (−0.035, 0.057)	**0.059** **(0.010, 0.109)**	0.016 (−0.033, 0.065)
Iron-rich	**1**	0.056 (0.015, 0.097)	0.076 (0.035, 0.117)	0.036 (−0.005, 0.077)	0.010 (−0.031, 0.051)	0.057 (0.016, 0.098)	0.039 (−0.002, 0.080)
	**2**	**0.078** **(0.018, 0.138)**	**0.088** **(0.027, 0.148)**	0.061 (−0.002, 0.123)	0.035 (−0.021, 0.090)	**0.071** **(0.011, 0.131)**	0.039 (−0.020, 0.099)
Low-iron	**1**	−0.032 (−0.079, 0.015)	−0.048 (−0.095, −0.001)	−0.049 (−0.096, −0.002)	−0.014 (−0.061, 0.033)	−0.001 (−0.048, 0.046)	−0.017 (−0.064, 0.030)
	**2**	−0.069 (−0.138, 0.001)	−0.050 (−0.120, 0.020)	**−0.095** **(−0.167, −0.023)**	−0.047 (−0.111, 0.017)	−0.023 (−0.093, 0.046)	−0.039 (−0.108, 0.030)
**Third trimester**							
Protein- and micronutrient-rich	**1**	0.017 (−0.017, 0.051)	0.013 (−0.021, 0.047)	0.028 (−0.006, 0.062)	0.003 (−0.031, 0.037)	0.020 (−0.014, 0.054)	0.006 (−0.028, 0.040)
	**2**	0.040 (−0.009, 0.089)	0.048 (−0.008, 0.091)	**0.063** **(0.012, 0.114)**	0.004 (−0.041, 0.050)	0.044 (−0.006, 0.093)	0.013 (−0.036, 0.062)
Tubers; fruits; baked food	**1**	0.004 (−0.036, 0.044)	−0.020 (−0.060, 0.019)	−0.022 (−0.061, 0.018)	0.004 (−0.036, 0.044)	0.028 (−0.012, 0.068)	0.011 (−0.028, 0.051)
	**2**	0.014 (−0.050, 0.078)	0.016 (−0.048, 0.080)	−0.019 (−0.086, 0.047)	0.006 (−0.053, 0.065)	0.047 (−0.017, 0.111)	−0.002 (−0.065, 0.061)
Low-iron	**1**	−0.037 (−0.083, 0.009)	−0.055 (−0.101, −0.009)	−0.039 (−0.085, 0.007)	−0.028 (−0.073, 0.018)	−0.014 (−0.059, 0.032)	−0.004 (−0.050, 0.042)
	**2**	−0.062 (−0.127, 0.003)	**−0.068** **(−0.134, −0.003)**	**−0.072** **(−0.140, −0.004)**	−0.049 (−0.109, 0.012)	−0.031 (−0.096, 0.035)	−0.009 (−0.073, 0.056)

Model 1: Unadjusted models; Model 2: Adjusting for the confounding factors such as maternal age, pre-pregnancy BMI, maternal occupation before pregnancy, family income, education level, maternal smoking, alcohol consumption, parity, anxiety, depression, and pregnancy complications. Bolding indicated *p*-values < 0.05.

**Table 3:** Association between different trajectory groups of dietary pattern during pregnancy and 36-month-old children’s neurodevelopment [β (95% CI)].

Maternal Dietary Pattern Trajectories	Total ASQ Score	Communication	Gross Motor	Fine Motor	Problem-Solving	Personal-Social
Protein- and micronutrient-rich						
Model 1	3.007 (−0.157, 6.171)	0.500 (−0.213, 1.213)	**0.725** **(0.070, 1.379)**	0.766 (−0.565, 2.097)	**0.897** **(0.041, 1.752)**	0.119 (−0.853, 1.092)
Model 2	**5.107** **(0.464, 9.750)**	0.939 (−0.109, 1.987)	**1.279** **(0.289, 2.269)**	0.994 (−0.837, 2.825)	**1.478** **(0.221, 2.735)**	0.417 (−1.004, 1.838)
Low-iron						
Model 1	−2.063 (−5.992, 1.866)	−0.040 (−0.921, 0.841)	−0.334 (−1.140, 0.473)	−0.882 (−2.536, 0.772)	−0.478 (−1.537, 0.581)	−0.329 (−1.538, 0.879)
Model 2	−1.032 (−6.670, 4.607)	−0.410 (−1.682, 0.861)	−0.112 (−1.316, 1.091)	−0.854 (−3.071, 1.363)	0.098 (−1.429, 1.625)	0.246 (−1.474, 1.967)

Model 1: Unadjusted models; Model 2: Adjusting for the confounding factors such as maternal age, pre-pregnancy BMI, maternal occupation before pregnancy, family income, education level, maternal smoking, alcohol consumption, parity, anxiety, depression, and pregnancy complications. Bolding indicated *p*-values < 0.05.

## Data Availability

The data are not publicly available due to privacy restrictions. If required, the data that support the findings of this study are available on request from the corresponding author.

## Supplementary Materials

The following supporting information can be downloaded at: https://www.mdpi.com/article/10.3390/nu16101530/s1, Figure S1: Directed acyclic graph to show the association between dietary pattern during pregnancy and children’s neurodevelopment; Table S1: Dietary patterns identified by PCA and factor loadings of food groups/items* for women in three trimesters of pregnancy; Table S2: Sensitivity analyses of the association between maternal dietary pattern scores during pregnancy and 36-month-old children’s neurodevelopment [β (95%CI)]; Table S3: Sensitivity analyses of the association between different trajectory groups of dietary pattern during pregnancy and 36-month-old children’s neurodevelopment [β (95%CI)].
