# Supplementary material for: Association between Dietary Patterns during Pregnancy and Children’s Neurodevelopment: A Birth Cohort Study
Source: Nutrients. 2024 May 19;16(10):1530. doi: 10.3390/nu16101530 (PMC11123670; doi:10.3390/nu16101530)
Supplement: Supplementary file 1 [file nutrients-16-01530-s001.zip › nutrients-2959276-supplementary.pdf]

Supplementary Figure S1 Directed acyclic graph to show the association between dietary pattern during pregnancy and children's neurodevelopment

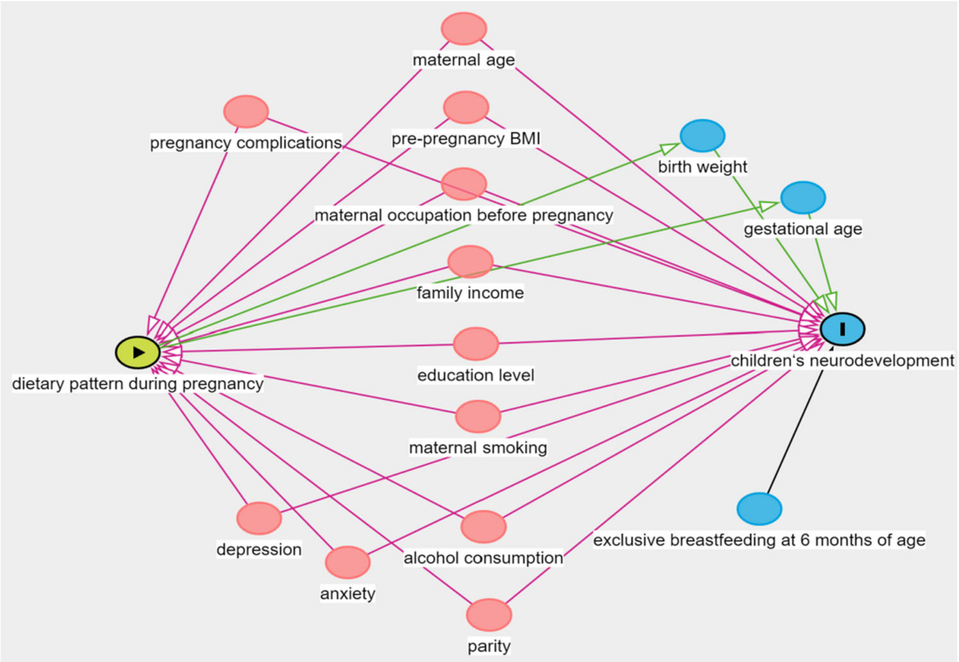

Supplementary Table S1 Dietary patterns identified by PCA and factor loadings of food groups/items* for women in three trimesters of pregnancy (n=1,423).

Food items	Maternal dietary pattern								
	The first trimester			The second trimester			The third trimester		
	Protein and micronutrient -rich	Low-iron	Pasta as the staple food	Protein and micronutrient -rich	Rich-iron	Low-iron	Protein and micronutrient -rich	Tubers; fruits; baked food	Low-iron
Rice	-	-	-0.625	-	-	-	-	-	-
Steam cooked pasta	-	-	0.587	-	-	-	-	-	-
Coarse cereals	0.358	-	-	0.387	-0.350	-	0.300	-0.457	-
Tubers	0.337	-	-	0.325	-	0.387	0.316	0.494	-
Fried dough foods	0.355	-0.387	-	-	0.442	-	-	-	-
Soybean-food	0.475	-	-	0.471	-	-	0.474	-	-
Soymilk	0.338	-	-	-	-	-	-	-	-
Fungal and algal food	0.508	-	-	0.487	-	-	0.488	-	-
Fresh vegetables	-	-	-0.333	-	-	-	-	-	0.330
Fresh fruits	-	0.367	-	-	-	0.637	-	0.673	-
Citrus fruits	-	-	-	0.353	-	0.421	0.319	0.640	-
Dry fruit	0.442	-	-	0.388	-	-	0.408	-	-
Nut	0.364	0.322	-	0.473	-	-	0.499	-	-
Animal offal	0.529	-	-	0.421	-	-0.300	0.459	-	-
Animal blood	0.548	-0.381	-	0.393	0.415	-0.319	0.410	-	-0.421
Shrimp	0.493	-	-	0.391	-	-	0.404	-	-
Fish	0.452	-	-	0.409	0.541	-	0.392	-	-
Mollusk	0.553	-0.493	-	0.428	0.562	-	0.349	-	-0.543
Shellfish	0.528	-0.549	-	0.325	-0.392	-	0.341	-	-0.590
Eggs	0.316	0.354	-	0.342	-	-	0.366	-	0.302
Dairy	0.307	-	-	0.323	-	-	0.325	-	-
Baked food	-	-	-	-	-	0.311	-	0.461	-
Meat	-	-	-	0.375	-	-	-	-	-

Factor loadings indicated food group contribution to each dietary pattern

Supplementary Table S2 Sensitivity analyses of the association between maternal dietary pattern scores during pregnancy and 36-month-old children's neurodevelopment [β (95%CI)]

Maternal dietary pattern		Total ASQ Score	Communication	Gross motor	Fine motor	Problem-solving	Personal-social
**First trimester**							
Protein and micronutrient-rich	Model3	**0.052 (0.002,0.101)**	0.049 (-0.001,0.099)	**0.053 (0.002,0.104)**	0.010 (-0.035,0.056)	0.049 (-0.001,0.098)	0.040 (-0.009,0.089)
	Model4	**0.057 (0.009,0.106)**	**0.052 (0.004,0.101)**	**0.058 (0.007,0.109)**	0.017 (-0.028,0.062)	**0.056 (0.008,0.105)**	0.037 (-0.011,0.085)
	Model5	**0.053 (0.006,0.101)**	**0.053 (0.006,0.100)**	**0.055 (0.007,0.104)**	0.010 (-0.034,0.054)	**0.052 (0.005,0.099)**	0.039 (-0.008,0.085)
Low-iron	Model3	-0.025 (-0.092,0.042)	-0.047 (-0.115,0.020)	-0.028 (-0.098,0.041)	0.020 (-0.043,0.082)	-0.054 (-0.121,0.013)	-0.009 (-0.075,0.057)
	Model4	-0.031 (-0.098,0.035)	-0.054 (-0.120,0.012)	-0.029 (-0.098,0.040)	0.017 (-0.045,0.078)	-0.066 (-0.132,0.001)	-0.008 (-0.074,0.057)
	Model5	-0.039 (-0.102,0.024)	-0.059 (-0.123,0.004)	-0.032 (-0.098,0.034)	0.015 (-0.044,0.074)	**-0.072 (-0.136,-0.009)**	-0.019 (-0.082,0.043)
Pasta as the staple food	Model3	-0.030 (-0.102,0.043)	-0.071 (-0.144,0.001)	**0.080 (0.005,0.154)**	-0.034 (-0.101,0.034)	-0.010 (-0.083,0.063)	-0.044 (-0.115,0.028)
	Model4	-0.032 (-0.103,0.040)	-0.069 (-0.041,0.002)	0.073 (-0.001,0.148)	-0.035 (-0.101,0.031)	-0.011 (-0.083,0.060)	-0.043 (-0.113,0.028)
	Model5	-0.038 (-0.105,0.030)	**-0.077 (-0.145,-0.010)**	0.058 (-0.012,0.128)	-0.028 (-0.091,0.035)	-0.026 (-0.093,0.042)	-0.044 (-0.111,0.023)
**Second trimester**							
Protein and micronutrient-	Model3	0.040	0.036	**0.062**	0.012	**0.053**	-0.001

rich		(-0.013,0.093)	(-0.017,0.090)	**(0.007,0.116)**	(-0.037,0.060)	**(0.001,0.106)**	(-0.052,0.052)
	Model4	0.047<br>(-0.005,0.099)	0.046<br>(-0.006,0.098)	**0.063**<br>**(0.009,0.117)**	0.014<br>(-0.034,0.062)	**0.057**<br>**(0.005,0.110)**	0.010<br>(-0.042,0.061)
	Model5	**0.050**<br>**(0.001,0.099)**	0.044<br>(-0.005,0.093)	**0.064**<br>**(0.013,0.115)**	0.015<br>(-0.031,0.060)	**0.060**<br>**(0.011,0.109)**	0.015<br>(-0.033,0.064)
Rich-iron	Model3	**0.082**<br>**(0.018,0.146)**	**0.091**<br>**(0.026,0.155)**	0.055<br>(-0.012,0.121)	0.043<br>(-0.016,0.102)	0.059<br>(-0.005,0.123)	0.054<br>(-0.009,0.117)
	Model4	**0.088**<br>**(0.025,0.151)**	**0.100**<br>**(0.036,0.163)**	0.057<br>(-0.009,0.123)	0.049<br>(-0.009,0.107)	**0.072**<br>**(0.009,0.136)**	0.044<br>(-0.018,0.107)
	Model5	**0.081**<br>**(0.021,0.140)**	**0.090**<br>**(0.030,0.150)**	0.060<br>(-0.002,0.122)	0.039<br>(-0.017,0.094)	**0.074**<br>**(0.014,0.134)**	0.039<br>(-0.020,0.099)
Low-iron	Model3	**-0.074**<br>**(-0.146,-0.001)**	-0.051<br>(-0.125,0.022)	**-0.099**<br>**(-0.173,-0.024)**	-0.045<br>(-0.112,0.022)	-0.032<br>(-0.105,0.041)	-0.046<br>(-0.117,0.025)
	Model4	-0.071<br>(-0.144,0.002)	-0.050<br>(-0.124,0.023)	**-0.102**<br>**(-0.178,-0.026)**	-0.044<br>(-0.111,0.023)	-0.025<br>(-0.098,0.049)	-0.044<br>(-0.116,0.028)
	Model5	-0.066<br>(-0.133,0.002)	-0.042<br>(-0.110,0.027)	**-0.095**<br>**(-0.165,-0.025)**	-0.046<br>(-0.110,0.017)	-0.020<br>(-0.088,0.049)	-0.039<br>(-0.106,0.029)
**Third trimester**							
Protein and micronutrient-rich	Model3	0.035<br>(-0.017,0.088)	0.037<br>(-0.015,0.090)	**0.066**<br>**(0.013,0.120)**	0.001<br>(-0.048,0.048)	0.044<br>(-0.009,0.096)	0.006<br>(-0.046,0.057)
	Model4	0.036<br>(-0.015,0.087)	0.042<br>(-0.010,0.093)	**0.059**<br>**(0.006,0.112)**	-0.001<br>(-0.047,0.047)	0.044<br>(-0.007,0.095)	0.008<br>(-0.042,0.058)
	Model5	0.036<br>(-0.013,0.084)	0.041<br>(-0.008,0.090)	**0.061**<br>**(0.011,0.112)**	0.001<br>(-0.045,0.046)	0.037<br>(-0.012,0.086)	0.011<br>(-0.037,0.060)
Tubers; fruits; baked food	Model3	0.011<br>(-0.056,0.079)	0.018<br>(-0.050,0.085)	-0.012<br>(-0.082,0.057)	-0.004<br>(-0.066,0.058)	0.032<br>(-0.035,0.099)	0.011<br>(-0.055,0.076)

	Model4	0.025 (-0.041,0.091)	0.277 (-0.039,0.094)	-0.007 (-0.076,0.063)	0.012 (-0.049,0.073)	0.050 (-0.017,0.116)	0.006 (-0.059,0.071)
	Model5	0.012 (-0.052,0.075)	0.016 (-0.048,0.080)	-0.020 (-0.086,0.047)	0.005 (-0.054,0.064)	0.040 (-0.023,0.104)	-0.002 (-0.065,0.060)
Low-iron	Model3	**-0.077 (-0.146,-0.008)**	**-0.078 (-0.147,-0.008)**	**-0.075 (-0.146,-0.004)**	-0.058 (-0.121,0.006)	-0.041 (-0.110,0.028)	-0.030 (-0.097,0.038)
	Model4	**-0.075 (-0.142,-0.007)**	**-0.075 (-0.143,-0.007)**	**-0.077 (-0.148,-0.007)**	-0.059 (-0.122,0.003)	-0.039 (-0.107,0.029)	-0.021 (-0.088,0.045)
	Model5	-0.054 (-0.119,0.010)	**-0.068 (-0.133,-0.003)**	**-0.065 (-0.133,-0.002)**	-0.042 (-0.102,0.018)	-0.026 (-0.091,0.040)	-0.003 (-0.067,0.061)

Bolding indicated p-values < 0.05;
Model3 Further adjusted for birth weight Z scores;
Model4 Further adjusted exclusive breastfeeding at 6 months of age;
Model5 Further adjusted infants’ complementary food within 6 months of age.

Supplementary Table S3 Sensitivity analyses of the association between different trajectory groups of dietary pattern during pregnancy and 36-month-old children's neurodevelopment [β (95%CI)]

Maternal dietary pattern trajectories	Total ASQ score	Communication	Gross motor	Fine motor	Problem-solving	Personal-social
Protein and micronutrient-rich						
Model3	4.062 (-0.910,9.035)	0.740 (-0.383,1.864)	**1.242 (0.190,2.295)**	0.822 (-1.123,2.767)	1.298 (-0.044,2.641)	-0.041 (-1.547,1.464)
Model4	4.515 (-0.354,9.384)	0.881 (-0.214,1.976)	**1.248 (0.207,2.290)**	0.830 (-1.071,2.731)	**1.382 (0.063,2.700)**	0.175 (-1.310,1.660)
Model5	**5.134 (0.486,9.783)**	0.941 (-0.109,1.991)	**1.279 (0.288,2.271)**	0.998 (-0.835,2.831)	**1.496 (0.238,2.753)**	0.421 (-1.002,1.843)
Low-iron						
Model3	-1.390 (-7.345,4.565)	-0.470 (-1.814,0.874)	-0.274 (-1.538,0.989)	-0.660 (-2.985,1.666)	-0.128 (-1.738,1.481)	0.143 (-1.657,1.942)
Model4	-0.857 (-6.679,4.964)	-0.415 (-1.723,0.893)	-0.108 (-1.355,1.140)	-0.638 (-2.906,1.630)	-0.074 (-1.651,1.504)	0.377 (-1.394,2.148)
Model5	-1.009 (-6.653,4.636)	-0.410 (-1.682,0.863)	-0.113 (-1.319,1.092)	-0.852 (-3.071,1.368)	0.116 (-1.413,1.644)	0.251 (-1.472,1.973)

Bolding indicated p-values < 0.05;

Model3 Further adjusted for birth weight Z scores;

Model4 Further adjusted exclusive breastfeeding at 6 months of age;

Model5 Further adjusted infants' complementary food within 6 months of age.
